# The Histidine Phosphocarrier Kinase/Phosphorylase from Bacillus Subtilis Is an Oligomer in Solution with a High Thermal Stability

**DOI:** 10.3390/ijms22063231

**Published:** 2021-03-22

**Authors:** José L. Neira, Ana Cámara-Artigas, José Ginés Hernández-Cifre, María Grazia Ortore

**Affiliations:** 1 IDIBE, Universidad Miguel Hernández, 03202 Alicante, Spain; 2 Instituto de Biocomputación y Física de Sistemas Complejos, Joint Units IQFR-CSIC-BIFI, and GBsC-CSIC-BIFI, Universidad de Zaragoza, 50009 Zaragoza, Spain; 3 Departamento de Química y Física, Research Center CIAIMBITAL, Universidad de Almería- ceiA3, 04120 Almería, Spain; acamara@ual.es; 4 Departamento de Química Física, Facultad de Química, Campus de Espinardo, Universidad de Murcia, 30100 Murcia, Spain; jghc@um.es; 5 Dipartimento DiSVA, Università Politecnica delle Marche, Via Brecce Bianche, 60131 Ancona, Italy; m.g.ortore@univpm.it

**Keywords:** circular dichroism, conformational stability, fluorescence, phosphorylation

## Abstract

The histidine phosphocarrier protein (HPr) kinase/phosphorylase (HPrK/P) modulates the phosphorylation state of the HPr protein, and it is involved in the use of carbon sources by Gram-positive bacteria. Its X-ray structure, as concluded from crystals of proteins from several species, is a hexamer; however, there are no studies about its conformational stability, and how its structure is modified by the pH. We have embarked on the conformational characterization of HPrK/P of *Bacillus subtilis* (bsHPrK/P) in solution by using several spectroscopic (namely, fluorescence and circular dichroism (CD)) and biophysical techniques (namely, small-angle X-ray-scattering (SAXS) and dynamic light-scattering (DLS)). bsHPrK/P was mainly a hexamer in solution at pH 7.0, in the presence of phosphate. The protein had a high conformational stability, with an apparent thermal denaturation midpoint of ~70 °C, at pH 7.0, as monitored by fluorescence and CD. The protein was very pH-sensitive, precipitated between pH 3.5 and 6.5; below pH 3.5, it had a molten-globule-like conformation; and it acquired a native-like structure in a narrow pH range (between pH 7.0 and 8.0). Guanidinium hydrochloride (GdmCl) denaturation occurred through an oligomeric intermediate. On the other hand, urea denaturation occurred as a single transition, in the range of concentrations between 1.8 and 18 µM, as detected by far-UV CD and fluorescence.

## 1. Introduction

The bacterial phosphoenolpyruvate (PEP)-dependent sugar phosphotransferase system (PTS) is a multi-protein transport chain responsible for carbohydrate uptake and transport through the cell wall. The PTS modulates the preferential use of carbon catabolite in bacteria in response to challenging metabolic and environmental conditions, but it is also involved in cell movement towards those carbon sources (chemotaxis), nitrogen metabolism, and the regulation of other metabolic pathways in both Gram-negative and Gram-positive [1,2,3,4,5,6]. Such PTS regulation occurs either by phosphorylating its target proteins, or alternatively, by interacting with them in a phosphorylation-dependent manner. The target proteins can be transporters, signal transduction proteins, catabolic enzymes, and, in many cases, transcriptional regulators [3,5,6,7]. The composition of PTS is similar in all species described to date: it is formed by several proteins which carry out phosphoryl-transfer steps, from PEP to the sugar-specific enzyme II permeases (EIIs). The first two proteins in the cascade are common to all PTS substrates (the so-called “general PTS proteins”): the phosphocarriers EI and HPr.

In enteric bacteria, the modulation of carbohydrate metabolism occurs through the EII proteins [8,9]; however, in Gram-positive bacteria, the regulation is focused on HPr. In these bacteria, HPr has two phosphorylation sites. In the PEP-dependent phosphotransfer process, phosphorylation of HPr at His15 occurs through the intervention of EI, and this phosphorylation is PEP-dependent, with the carbohydrate as the final recipient of the phosphate. In addition, HPr is involved in regulatory, ATP-dependent phosphorylation at Ser46 by the HPr kinase/phophorylase (HPrK/P), which is a general sensor for free phosphate, ATP and glycolytic intermediates [3,5,7,10,11,12,13,14,15,16,17,18]; the kinase activity is activated by fructose-1,6-biphopshate and inhibited by free phosphate [17]. The X-ray structures of HprK/P from several species show that the protein is a hexamer, where each monomer has a mixed α/β structure [19,20,21,22]. However, except for early studies characterizing its activity [17,18,23] and its oligomeric state in solution, there is no description of the conformational stability of HPrK/P in aqueous solution, no clue as to its biophysical properties, and no insight into how its structure in solution changes as the pH is modified.

In this work, we used the HPrK/P from *Bacillus subtilis*, bsHPrK/P, as a model to elucidate the structural and conformational preferences of this kind of protein in a wide pH range. We chose this protein as a model because it is expressed in high amounts, easily purified and there are no detailed structural studies of it; in fact, there is no crystal structure [19,20,21,22]. Our results show that the protein was a hexamer in solution by using DLS and SAXS. The protein precipitated between pH 3.5 and 6.5 and its stability was higher at pH 7.0, acquiring a native-like structure within a narrow pH range (from 7.0 to 8.0). Urea denaturation occurred with a single transition, in the range of concentrations between 1.8 and 18 µM, indicating that dissociation and unfolding occurred concomitantly. However, GdmCl denaturation showed the presence of an oligomeric intermediate, indicating that the presence of ionic strength may affect the folding pathway. 

## 2. Results

### 2.1. bsHPrK/P Acquired a Native-Like Structure in a Narrow pH Range 

We are aiming, in the future, to study the binding of bsHPrK/P with HPr proteins of several species, with the goal of designing peptides (or protein fragments) capable of hampering such interaction; then, we must determine which pH range enables the study of binding, as the HPr proteins have a narrow pH range (>7.0) in which they are very stable and they acquire a native-like conformation. Furthermore, to measure the conformational stability of bsHPrK/P, we must first determine in which pH range it acquired a native-like structure. To that end, several spectroscopic and biophysical probes were used, namely intrinsic fluorescence, ANS fluorescence and far-UV CD [24]. The use of the whole set of techniques gives us complementary information on the different structural features of bsHPrK/P. We used intrinsic fluorescence to monitor the changes in the tertiary structure of the protein, around its single tryptophan (Trp235) and five tyrosine residues (Tyr43, Tyr46, Tyr47, Tyr145 and Tyr241). We used ANS fluorescence to follow the accessibility of solvent-exposed hydrophobic patches and to detect the presence of partially folded species [25]. Finally we carried out far-UV CD experiments to monitor changes in its secondary structure.

#### 2.1.1. Fluorescence

*(1) Intrinsic fluorescence and thermal denaturation—*The fluorescence emission spectra of bsHPrK/P showed a maximum of around 340 nm at physiological pH (Figure S1A). Thus, the fluorescence spectrum was dominated by the emission of Trp235, which appeared, from the value of the maximum wavelength, to be partially buried. We followed the fluorescence intensity value and calculated <λ>, which is called the average energy (Section 4.7.). This parameter gives a measurement of the overall intensity of the spectrum (a sort of integral value) and, thus, it is less affected by any pipetting error than the analysis using a single-intensity value at a particular wavelength. By excitation at 280 nm, we excite tyrosine residues and the sole tryptophan; by excitation at 295 nm, we follow only the tryptophan residue. Thus, by using both wavelengths we can obtain complementary information and we can see whether the environment around the five tyrosine residues (Tyr43, Tyr46, Tyr47, Tyr145 and Tyr241) changes concomitantly with that of the sole tryptophan (Trp235).

**Figure 1:**
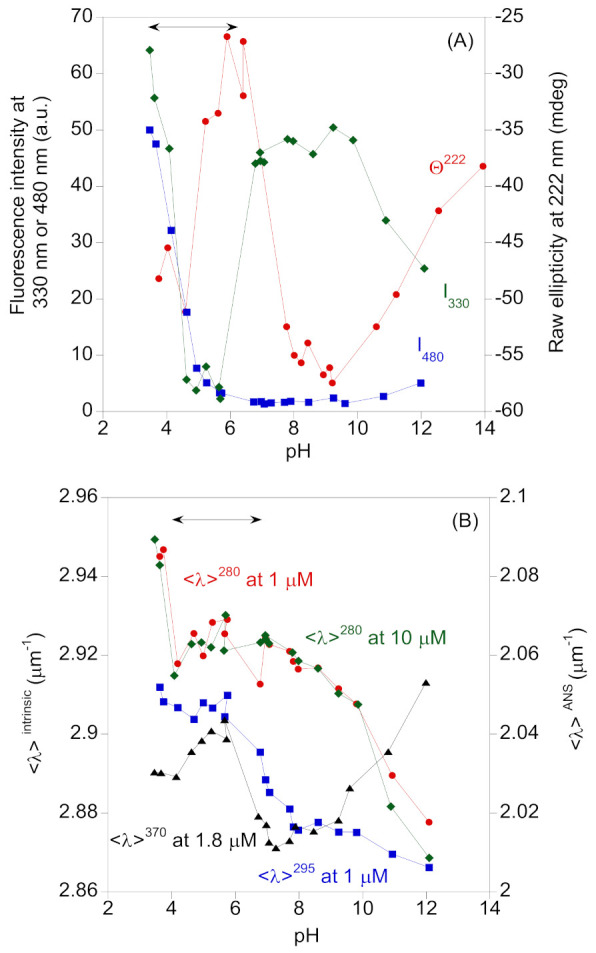
pH-induced structural changes in bsHPrK/P, followed by spectroscopic techniques: (**A**) Changes in the intrinsic fluorescence intensity emission at 330 nm, after excitation at 280 nm (left axis); scaled-up ANS fluorescence emission at 480 nm (left axis); and raw ellipticity at 222 nm (right axis). Experiments were carried out at 1.8 µM of protein concentration (for all techniques) and 25 °C. (**B**) Changes in the <λ>, after excitation either at 280 or 295 nm (left axis), after excitation at 370 nm (right axis) at different protomer concentrations as indicated in the figure. The lines are drawn to guide the eye. The experiments with each technique were repeated three times, but only a complete, single titration is shown for each technique. The double-arrow line at the top in both panels indicates the pH range where precipitation of the protein was observed. Samples at those pH values were centrifuged to remove the precipitate.

Fluorescence intensity at 330 nm (close to the maximum wavelength, after excitation at 280 nm) at any of the explored concentrations (1, 1.8, 10 and 15 µM) showed the same four regions: (i) a gradual decrease in acidic pH values (until pH 3.5); (ii) an abrupt diminution between pH 3.5 and 6.5 (where we observed precipitation in the samples, close to the p*I* of the protein without the His-tag (5.10)); (iii) a constant value of the fluorescence intensity between pH 7.0 and 10.0; (iv) a stepwise reduction from pH 10 (Figure 1A). The same was observed after excitation at 295 nm at the four explored concentrations. The <λ> showed a similar behavior, although the scattering of experimental data between pH 3.5 and 6.5 was smaller than that seen by observing the intensity, and it had lower basic pH values, suggesting that at pH values larger than 8.0, fluorescence started decreasing (Figure 1B); furthermore, as <λ> followed a similar pattern at different concentrations (1.8 and 10 µM are shown in Figure 1B), the pH denaturation behavior was protein-concentration-independent. This finding indicates that no dissociation of the self-associated species occurs (Section 2.2). The tendency of <λ>^295^ was slightly different to that of <λ>^280^, especially in: (i) the region between pH 3.5 and 6.5, where precipitation was observed (a decrease of <λ>^295^ occurred); (ii) at basic pH values, where a shorter decrease in the <λ>^295^ values was observed when compared to the <λ>^280^ values. This indicates that subtle conformational changes around tyrosines occur between pH 7.0 and 9.0. Both <λ> values at basic pH values show another titration, probably due to tyrosine residues [26,27]. These findings indicate that the tryptophan was more sensitive to the precipitation, and less sensitive to the basic titration of tyrosines. Furthermore, these results pinpoint the importance of monitoring fluorescence at the two excitation wavelengths. It is important to indicate that, in terms of absolute value, the changes in <λ> are small (because it is an integral of the intensity over the whole spectrum) but they mirror, except for the lower data scattering, the tendency observed when following the fluorescence intensity (Figure 1A).

The above results from fluorescence suggest that the protein had a well-folded, native-like structure between approximately pH 7.0 and 8.0. To further test those results, we carried out thermal denaturations at pH 3.0, 12.0 (at a concentration of 1 µM of protomer); and pH 7.0 (at 1 and 14 µM), 7.3, 7.7 and 8.0 (at 14 µM). We did not observe any sigmoidal behavior at the acidic and basic pH values, suggesting that the protein at the both extreme of pH values did not contain any well-folded structure (Figure S2A); on the other hand, we did observe a sigmoidal behavior at the rest of the pH values explored (between pH 7.0 and 8.0). We did not observe any variation in the two concentrations explored at pH 7.0 (1 and 14 µM), indicating that, in this concentration range, the determined apparent *T*_m_ was not protein concentration-dependent. The apparent *T*_m_ values (Figure S2B) in °C (errors within parenthesis are fitting errors to Equation (4) by taking Equation (5) into account, Section 4.7) are: 71.08 ± 0.04 (at pH 7.0 at the two concentrations); 58.85 ± 0.04 (at pH 7.3); 56.35 ± 0.04 (at pH 7.7) and 57.42 ± 0.09 (at pH 8.0). This shows that the protein seemed to be more stable at pH 7.0 and it was very pH-sensitive. We do not know the reasons for the large variation in the apparent *T*_m_ in such a short pH interval, but they could be related to: (i) any conformational change occurring around Trp235 (since the thermal transition was observed by excitations at 280 and 295 nm), or, alternatively, (ii) a larger irreversibility occurring at lower pH values (closer to the pH interval where precipitation was observed). Furthermore, as a single transition was observed at all pH values, we can conclude that: (i) thermal oligomer dissociation (Section 2.2) and unfolding of a monomer occurred concomitantly; (ii) fluorescence was spectroscopically silent to dissociation; or (iii) dissociation and oligomerization had a similar, but not identical, *T*_m_, as monitored by fluorescence.

*(2) Quenching of the intrinsic fluorescence*—To measure the accessibility of the Trp and Tyr, we carried out quenching experiments with KI and acrylamide (Table 1). We used those quenching agents as they are those more frequently used to monitor the solvent-accessibility of fluorescent residues, because one of them has a negative charge (KI) and the other is non-polar (acrylamide); acrylamide could have more easy access to hydrophobic patches (see, for instance, [23]). These experiments address the solvent-accessibility of tryptophan and tyrosine to the quencher agents to see if such accessibility, and, therefore, the tertiary environment, changes when the pH or solution conditions are modified. We carried out the experiments at (i) pH values of 7.0 and 8.0, where the protein had a fairly constant value of the fluorescence intensity; (ii) 5 M GdmCl, pH 7.0, where the protein was unfolded (see Section 2.3.2). We did not carry out experiments at acidic pH due to the presence of aggregation and the absence of structure (see Section 2.1.2 and Section 2.2, and the ANS experiments). The *K*_sv_ is the collisional Stern–Volmer constant, and it provides a measurement of the solvent-accessibility of fluorescent residues. A larger value of *K*_sv_ indicates a higher number of solvent-exposed fluorescent residues. Under both quenching conditions, the *K*_sv_ values were, in general, larger at 280 than at 295 nm, because at the first wavelength we excited both Trp235 and the five tyrosine residues, whereas at 295 nm we only excited the single tryptophan; the differences, however, were larger in acrylamide than in KI. The sole exception was acrylamide quenching at pH 7.0, where the *K*_sv_ value at 280 was slightly smaller than at 295 nm (Table 1). For both quenchers, the values were larger when the protein was in 5 M GdmCl than under any other condition, suggesting that all bsHPrK/P’s tyrosine and tryptophan residues were fully solvent-accessible (and then the protein is unfolded; see Section 2.3).

In acrylamide, at pH 7.0 and 8.0, the *K*_sv_ values obtained by excitation at 295 nm were similar, indicating that although the protein had a different conformation at basic pH and an apparently lower *T*_m_ (Figure S2A), the solvent-accessibility of Trp235 did not change substantially in that pH range. The difference observed between the *K*_sv_ values determined at pH 7.0 and 8.0 at 280 nm could suggest that the environment around tyrosine residues is changing (as further suggested by the difference in the behavior of <λ>^295^ and <λ>^280^, Figure 1B).

In KI, except at 5 M GdmCl, where the *K*_sv_ value was much larger than at the rest of the other conditions, the Stern–Volmer constants did not show a large variation at any pH. This difference to the behavior observed in acrylamide could be due to the negative charge of the iodide.

It is important to note that quenching experiments in previous works were carried out at pH 8.2 (20 mM Hepes buffer) in the absence or presence of 6 M GdmCl for bsHPrK/P [23]. Although fitting of the data was carried out with a modified Stern–Volmer equation (and not the simplest, original Equation (1) or (2), Section 4.3.4), the values in such work qualitatively agree with those in Table 1: the *K*_sv_ values were larger in the presence of the denaturing agent than under any other condition. 

In conclusion, the intrinsic fluorescence changes as the pH was modified indicate that: (i) the protein had solvent-exposed tryptophan and tyrosine residues at 5 M GdmCl; (ii) under the other conditions, both types of aromatic residues appeared partially buried. 

*(3) ANS-binding*—An acidic transition for the ANS fluorescence intensity at 480 nm upon pH variation was observed (Figure 1A). We chose this wavelength to monitor the changes in the pH denaturation, because it is the maximum wavelength at acidic pHs (when there is binding between the protein and the probe). This transition was finished at pH 6.0, but we could not determine its p*K*_a_ value because of the absence of acidic baseline. This finding shows that this acidic transition, which partly overlaps with the observed protein precipitation, must leave some solvent-exposed hydrophobic patches. At basic pH values, another transition was observed, mirroring that detected by intrinsic fluorescence (Figure 1A). On the other hand, the variation of <λ> from ANS experiments more clearly reports that, for the intensity at 480 nm, the precipitation is between pH 3.5 and 6.5 (there was an increase in the <λ>^370^), and mirrors the behavior followed by the variation in <λ>^295^ (Figure 1B).

#### 2.1.2. Far-UV CD

*(1) Far-UV CD spectra*—The CD spectrum of bsHPrK/P at pH 7.0 showed minima at 210 and 220 nm (Figure S1B), although that at 220 nm was slightly more intense than the one at 208 nm. It is important to indicate that, at this latter wavelength, also the aromatic residues absorb [28,29,30,31] and there are 1 tryptophan, 5 tyrosine and 8 phenylalanine residues in the sequence of bsHPrK/P.

In the pH titrations, followed by the raw ellipticity at 222 nm (Θ^222^) acquired at a protomer concentration of 3.6 µM, we observed (Figure 1A): (i) the precipitation occurring between pH 3.5 and 6.5 (a decrease in the Θ^222^); (ii) a region between pH 8.0 and 9.0 where the Θ^222^ remained basically unaltered; (iii) a stepwise decrease in the Θ^222^ (in absolute value) at basic pH values. We followed the Θ^222^ to monitor changes in the pH, due to: (i) the presence of a minimum close at this wavelength in the spectrum, acquired at physiological pH (Figure S1B); (ii) the better signal-to-noiseratio of the spectrum at this wavelength when compared to that at 210 nm; (iii) the fact that, at this wavelength, the α-helix structure absorbs [28,29,30,31]. 

Taking the results of the three probes, it can be concluded that the pH-denaturation of the protein, followed by intrinsic fluorescence and CD, was basically the same (although, in one of them, the probe showed a decrease in the property and it had an increase in the other) with some differences in the behavior of both probes around physiological pH. The Θ^222^ and the fluorescence intensity at 330 nm (Figure 1A) showed constant values in a pH interval from around 8.0 to 9.0, and they changed at basic pH values, while they had a large variation when the protein precipitated. However, the Θ^222^ changed between pH 7.0 and 8.0, while the fluorescence intensity remained constant (Figure 1A). On the other hand, the ANS intensity did not seem to monitor the precipitation occurring between pH 3.5 and 6.5, since it was masked by the titration at acidic pHs, and in the interval between pH 6.5 and 8.5, there was no large variation in the ANS intensity; at basic pH values, the ANS intensity changed as the Θ^222^ and the fluorescence intensity at 330 nm did. 

*(2) Thermal denaturations*—We also carried out thermal denaturations at different pH values, followed by far-UV CD (Figure S3A). (i) It had been observed in fluorescence that, at acidic and basic pH, there were no thermal denaturations; (ii) the results of the pH titrations followed by CD mirrored those obtained by fluorescence; we explored thermal denaturations followed by CD at pH 7.6 and 8.0 (at 3.6 µM), and 7.0, 7.6 and 8.0 (at 1.8 µM of protomer). It is important to note that pH 7.0 is close to the pH-transition observed at Θ^222^ (Figure 1A); however, we do not know whether this transition is due to the subtle changes already detected in the aromatic residues (as we observed in the fluorescence by following the changes in <λ>^295^ and <λ>^280^, Section 2.1.1, or in the quenching results) or to large conformational changes. As we did not observe any large changes in the intrinsic fluorescence parameters monitored, or in the ANS, at this pH, we favored the first explanation. 

At the highest protomer concentration, we observed two transitions at pH 7.6 with apparent *T*_m_ values of (errors within parenthesis are fitting errors to Equation (4), by taking Equation (5), Section 4.7) 62.1 ± 0.5 °C and 72.7 ± 0.7 °C, whereas, at pH 8.0, we observed a transition with an apparent midpoint of 44.6 ± 0.2 °C. On the other hand, at the lowest protomer concentration (Figure S3B), we observed, at pH 7.0, a single, sigmoidal transition with a midpoint of 61.3 ± 0.2 °C (indicating that, at this pH, the protein has a well-folded conformation) and, at pH 8.0, another single sigmoidal with 47.8 ± 0.9 °C, and at pH 7.6 we observed two transitions: 57.94 ± 0.06 and 73.7 ± 0.9 °C. This behavior was similar to that observed at the lowest concentration for both values. At pH 7.6, the apparent thermal denaturation midpoint of the first transition was protein-concentration-dependent, as expected for an oligomer dissociation [32,33,34,35,36], whereas the second transition, within the fitting error, had the same *T*_m_ value at both protein concentrations, suggesting that it reports monomer unfolding. This is the sole pH where dissociation and unfolding were clearly observed as separate events. The similarity of the values of both apparent midpoints suggests that both processes could overlap at the other pH values, resulting in an apparent, single, observable transition. Furthermore, the thermal denaturation midpoint at pH 8.0 also seemed to be concentration-dependent, but, in this case, the highest apparent *T*_m_ was that observed at the lowest protein concentration (Figure S3), conversely to what would be anticipated; this unexpected result is probably due to: (i) the high irreversibility of the dissociation and unfolding processes at basic pH values; (ii) the possible similarity of the values of the midpoints of d at this pH. Due to the presence of two equilibria (the unfolding of the protein and the formation of non-specific self-associated species), the higher concentration of protein favors the second equilibrium shifting the first one (protein unfolding) and resulting in a lower apparent *T*_m_ for the highest concentration. The lower *T*_m_ value at pH 8.0 (compared to those at other pH values) could be due to the irreversibility which could be favored at basic pH values (Section 3.2) and the fact that this pH value is very close to the beginning of the titration of the tyrosine residues (as indicated by the variation in <λ>, Figure 1B).

Therefore, the thermal denaturation of HPrK/P, as monitored by far-UV CD, was not two-state, and shows the presence of dissociation and unfolding events.

### 2.2. bsHPrK/P Was an Oligomeric Species in Solution at Physiological pH 

The size and the oligomeric state of bsHPrK/P in solution at physiological pH were also addressed. Previous studies have suggested, by using analytical ultracentrifugation and size-exclusion chromatography [23], that the protein in solution is probably an octamer, although the posterior resolution of the X-ray structures of different HPrK/P members of the family showed the presence of hexamers [19,20,21,22]. Evidence from the far-UV CD thermal denaturations at pH 7.6 indicates that the protein was an oligomer, because it dissociated before melting of the monomer occurred (Figure S3) (and also from the GdmCl denaturations, Section 2.3.2). To elucidate the self-association state of the protein, we used DLS and SAXS, which measure different features of a possible self-association species. We carried out the experiments in the absence of a buffer (i.e., in pure water, where a pH of 7.2–7.4 was measured, depending on the concentration of the protein stock) and in the presence of a 50 mM sodium phosphate buffer (pH 7.0) to elucidate whether the possible self-associating properties of the protein were buffer-dependent at different protein concentrations. We suspected that there could be an effect of the buffer through inspection of the solved X-ray structures of other kinases (Discussion, Section 3.1).

**Figure 2:**
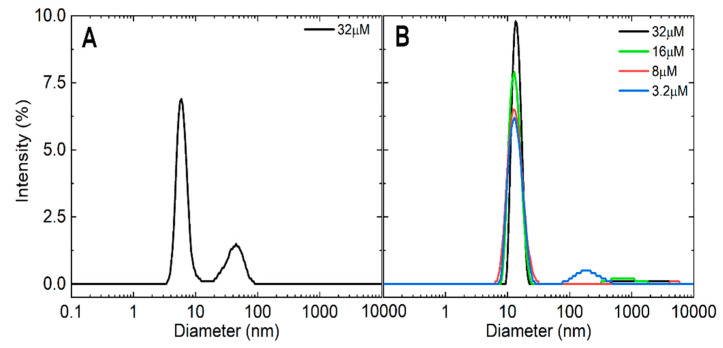
Self-association states of bsHPrK/P monitored by dynamic light-scattering (DLS): The DLS intensity distribution profiles of bsHPrP/K in pure water at 32 µM (**A**) and in 50 mM sodium phosphate buffer (pH 7.0) at different concentrations (**B**). Experiments were carried out at 25 °C.

DLS experiments were conducted at 32 µM in water and in 50 mM sodium phosphate buffer (pH 7.0) in the concentration range 3.2–32 µM (Figure 2). The hydrodynamic radius of bsHPrK/P in water showed two peaks in the intensity distribution: the first peak was monodisperse and corresponded to 99.9% of the protein in the solution with *R*_h_ = 2.9 ± 0.4 nm, and a second peak in aggregated species with *R*_h_ = 21.4 ± 4.4 nm (Figure 2A). The empirical MW of the mayor peak was 41 ± 6 kDa, which suggests the presence of a monomer of the HPrK/P in the solution (the theoretical molecular weight of the protein was 36.6 kDa, including the His-tag of the protein). In the presence of sodium phosphate, the protein showed an increase in the hydrodynamic radius, with an average value *R*_h_ = 6.4 ± 1.8 nm, which accounts for almost the 100% of the protein in the solution; only a small aggregate peak was visible in the intensity distribution plot (Figure 2B). The *R*_h_ value did not change upon dilution and the empirical MW was 264 ± 80 kDa. Taking the protein molecular weight (36.6 kDa) into account, the MW from the DLS measurements in sodium phosphate suggests the presence of either an octamer, as reported previously by AUC [23], or a hexamer. The octamer was considered the main self-associated species before the structures of the other homologous proteins were known. Indeed, the crystallographic structures of other members of the family show a hexamer with a propeller shape as stable oligomer [19,20,21,22]. Knowing the structure, the overall shape of this hexamer deviates from an ideal compact globular form, and then some deviation in the value obtained for the hydrodynamic radius of this oligomer is expected. In agreement with the available structural information in the PDB, the hexameric species are the most probable species in the solution in bsHPrK/P. At pH values lower than 7.0, bsHPrK/P is aggregated by forming oligomers of different MWs, which can be oligomers of the hexamers (dimer of the hexamer, dodecamer, MW of 420 kDa, and even a dimer of the dodecamer, with MW of 840 kDa). Experiments in the presence of Tris (50 mM, pH 7.2) were also carried out, and we observed the formation of self-associated species.

**Figure 3:**
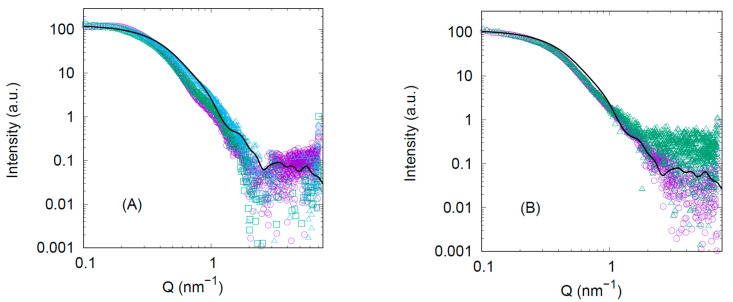
Self-association states of bsHPrK/P monitored by small-angle x-ray scattering (SAXS): (**A**) SAXS curves obtained in water and normalized according to their nominal concentration (39, 18 and 8 µM for violet circles, green squares and cyan triangles, respectively), and the theoretical curve (black line) for the X-ray structure (PDB entry: 1KNX). (**B**) SAXS curves obtained in 50 mM sodium phosphate buffer (pH 7.0) and normalized according to their nominal concentration (35 and 8 µM for violet circles and green triangles, respectively), and the same theoretical curve reported in panel (**A**). Experiments were carried out at 25 °C.

The SAXS analyses show that, in water, as the sample was more diluted, there were variations in the higher Q-range (Figure 3A); these variations could result from the presence of self-associated species (which could be those observed in DLS, Figure 2A, with *R*_h_ = 21.4 ± 4.4). SAXS curves in water did not show evidence of the monomer, because SAXS is proportional to molecule volumes; hence, if we have hexamers and monomers, we mainly see the hexamers. For the two protein concentrations explored in the presence of sodium phosphate (50 mM, pH 7.0), the normalized curves were the same, and thus there was no evidence of other species (Figure 3B). The Kratky plots under the different conditions are shown in the Supplemental Information (Figure S4); the bell shape of the Kratky plot representation confirms the compact structure of the protein in solution. The calculated gyration radius of the X-ray structure (PDB entry: 1KNX; as there is no X-ray structure for bsHPrK/P, this structure corresponds to the HPrK/P of *Mycoplasma pneumoniae*) for the hexamer is 4.4 ± 0.2 nm (black lines in Figure 3). In water, the gyration radius was 4.8 ± 0.2 nm, and in sodium phosphate it was 5.0 ± 0.2 nm. Both values were similar, within the margin of error, although that in the presence of sodium phosphate was slightly higher; we suggest that such a small difference might indicate the presence of a very low amount of other higher MW species.

In conclusion, DLS experiments in pure water (pH 7.2–7.4) indicate the presence of monomeric species, and it was in the presence of sodium phosphate when the hexamer appeared, mainly populated with other self-associated species.

### 2.3. Conformational Stability of bsHPrK/P 

The conformational stability of HPrK/P was measured using chemical denaturants, monitored with fluorescence (intrinsic and ANS) and far-UV CD at pH 7.0 (50 mM sodium phosphate buffer). We used this pH for the following reasons: (i) it had a value where fluorescence and CD indicate a co-operative thermal denaturation with a maximum apparent *T*_m_ (Section 2.1); (ii) it was close to the physiological pH; and (iii) it comes before the last transition (that of tyrosine residues) observed by the three probes used (Figure 1).

**Figure 4:**
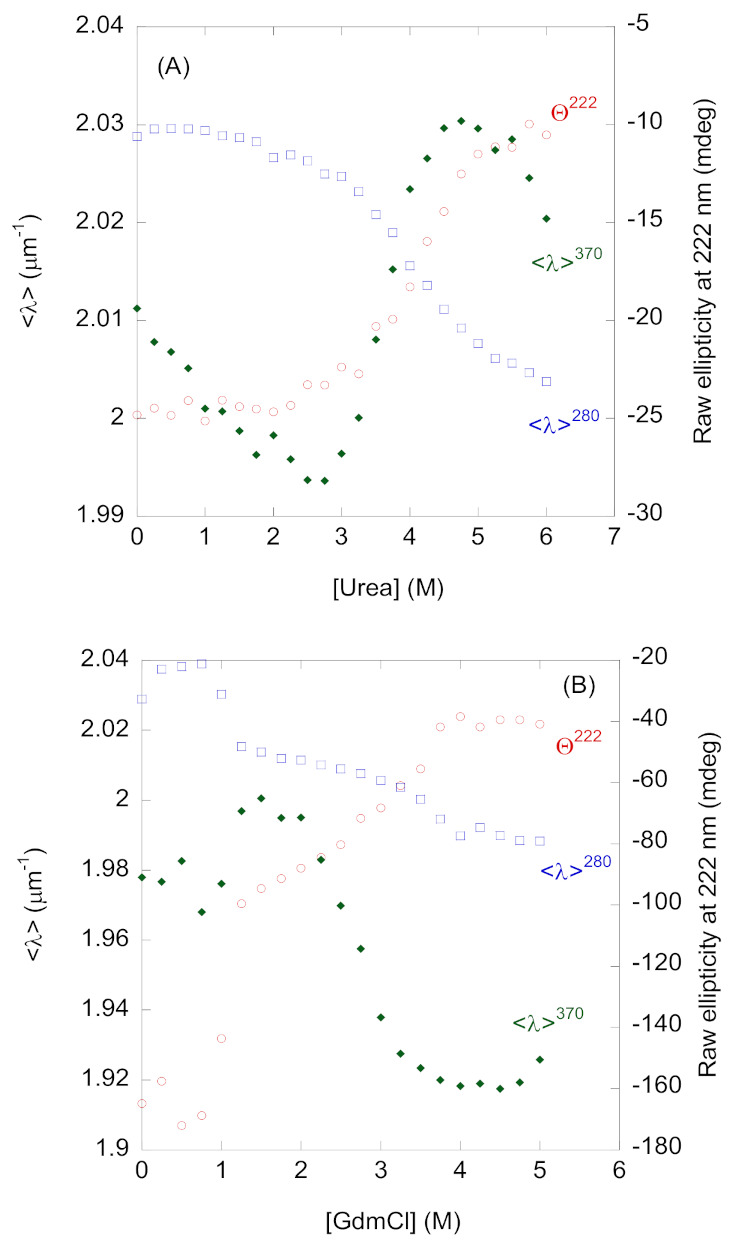
Chemical denaturations of bsHPrK/P followed by spectroscopic techniques: (**A**) Urea denaturations followed by the intrinsic fluorescence monitored by the <λ>, after excitation at 280 nm (<λ>^280^); by ANS fluorescence monitored by the <λ> (both on the left axis) (<λ>^370^); the raw ellipticity at 222 nm (Θ^222^) (right axis). The values of <λ>^280^ were scaled (by a factor of 1.44) to allow for a comparison with those of ANS. (**B**) GdmCl denaturations followed by the intrinsic fluorescence monitored by the <λ>, after excitation at 280 nm (<λ>^280^); by the ANS fluorescence monitored by the <λ> (both on the left axis) (<λ>^370^); the raw ellipticity at 222 nm (Θ^222^) (right axis). Protein concentration used in the GdmCl experiments followed by CD and fluorescence was 12.6 µM.

Chemical denaturations were carried out at different protein concentrations (Table 2) to elucidate whether there were two transitions (as was observed in the thermal denaturations followed by far-UV CD). Furthermore, we carried out experiments with two chemical denaturants (urea and GdmCl) to elucidate whether the ionic strength affected unfolding. For both denaturants, unfolding reactions were always irreversible, followed either by far-UV CD or intrinsic fluorescence (Figure S5). The unfolding reactions were irreversible, because the curves obtained from unfolding (Figure 4) and refolding (Figure S5) were not equal: there was a hysteresis behavior, and both folding and unfolding reactions did not follow the same path. Therefore, we cannot obtain a measurement of ∆*G* in the absence of a chemical denaturant. In the following paragraphs, we report the [denaturant]_1/2_- and *m*-values for each denaturant, trying to obtain qualitative conclusions on the protein-folding behavior that occurs in the presence of both chemicals.

#### 2.3.1. Urea Denaturations

All urea denaturations at different protein concentrations, using the three probes, showed a single sigmoidal transition (Figure 4A) with a midpoint, [urea]_1/2_ at around 4.0 M. The sole exception was the denaturation followed by ANS, where the [urea]_1/2-_value was smaller than those reported by the other techniques (Table 2). This result indicates that urea denaturation is not a two-state process [37]. None of the experiments at the different protein concentrations showed concentration-dependence, having a similar [urea]_1/2-_ and *m*- values within the margin of error (Table 2).

#### 2.3.2. GdmCl Denaturations.

Conversely to what was observed in the urea denaturations, the GdmCl denaturations at any of the explored bsHPrK/P concentrations showed (Figure 4B): (i) at least two transitions, one occurring at low GdmCl concentrations, which seemed to finish at 1 M GdmCl, and the other occurring at ~3.2 M (from intrinsic fluorescence and far-UV CD data) or 2.5 M (from ANS titrations); (ii) a protein-concentration-dependence in the midpoint of the second transition, which was more clearly visible after excitation at 295 nm (Figure S6B, Table 2). These latter results indicate that the second transition likely involved oligomer dissociation and monomer unfolding. Furthermore, our findings also indicate that the populated intermediate after the first transition was oligomeric. Since we do not know the order of the self-associated species at 1 M GdmCl (when the second transition seemed to start), we cannot rule out that the second transition involved the dissociation of trimers or tetramers from the initial most-populated hexamers. The first transition did not seem to be protein-concentration-dependent, as suggested by the similar behavior of the <λ> (after excitation at 280 nm) at two concentrations (Figure S6), although we cannot rule out a partial oligomer dissociation due to the scattering of the <λ> data. Thus, the first transition might involve a partial oligomer dissociation or, alternatively, conformational rearrangements in the hexameric species. Interestingly enough, the transition at low GdmCl concentration was more clearly observed by excitation at 280 than at 295 nm (Figure S6B), indicating that Trp235 was not mainly involved in the conformational rearrangement and/or partial dissociation. This is in contrast with what was observed for the second transition (Table 2).

We explored the protein behavior in the range from 0 to 0.75 M GdmCl concentrations. To find out whether the protein was folded in that denaturant concentration range, thermal denaturations, followed by intrinsic and ANS fluorescence, were carried out. All the thermal denaturations in the presence of any amount of GdmCl were irreversible. The results are shown in Table 3, and they indicate that: (i) the protein was folded in that GdmCl concentration range (otherwise we would not observe any transition); (ii) the apparent *T*_m_ decreased as the concentration of denaturant was raised (as the protein is becoming more unstable). We could not obtain any conclusion from a comparison of the values of the thermal denaturation midpoints (intrinsic or ANS fluorescence) at the same GdmCl concentration. Thus, in that interval of GdmCl concentrations, the protein was folded until at least 1 M GdmCl, but we could not define its oligomerization state.

## 3. Discussion

### 3.1. The Oligomeric State of bsHPrK/P in Solution

The biophysical and conformational characterization of bsHprK/P is an essential first step in deciphering its protein interactome, as well as in finding biochemical features that could mediate its interaction with other molecules. To the best of our knowledge, this is the first description of the conformational features of isolated bsHPrK/P in solution.

**Figure 5:**
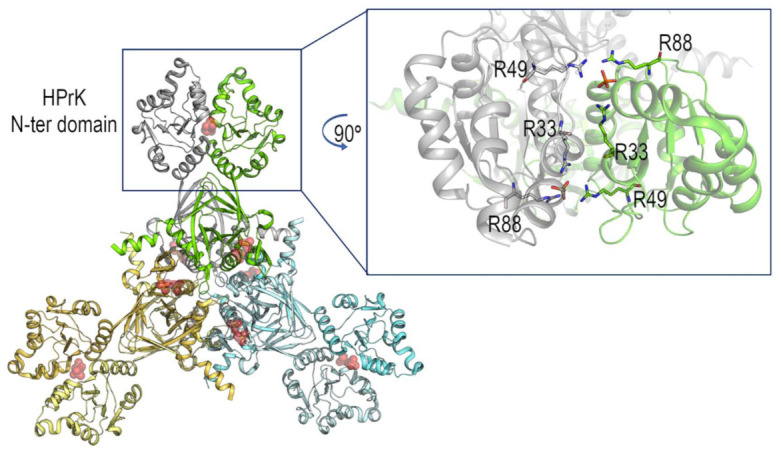
Structure of HPrK/P of *Staphylococcus xylosus*. (Left side) Hexamer of the HPrK/P from *Staphylococcus xylosus* (PDB entry 1KO7). The dimer in the asymmetric unit generates the hexamer through the symmetry operators. At the N-terminal domain of the HPrK/P, the interface between the dimer is stabilized by a salt bridge. (Right side) Detail of the salt bridge between the phosphate ions located at the interface and the arginine residues (shown in sticks). To facilitate the view of these interactions, the molecule has been rotated 90°. The figure was produced with Pymol [39].

All the X-ray structures of members of the HPrK/P family form hexamers [19,20,21,22], although the HPrK/P from *Enteroccus faecalis* has been shown to function as a dimer [17] and that of *B. subtilis* has been suggested to be an octamer by using AUC in solution [23]. Our results suggest that, in pure water, the monomer of bsHPrK/P is the main species (as concluded from DLS (Figure 2A)). The SAXS results were not conclusive, because the theoretically calculated gyration radius from the X-ray structure (PDB entry: 1KNX, corresponding to the HPrK/P of *Mycoplasma pneumoniae*) was similar to that measured in pure water (pH 7.2) and sodium phosphate buffer (pH 7.0). However, in the sodium phosphate buffer, the protein seemed to mainly populate the hexameric species (as suggested by DLS and SAXS). We wondered what triggered the formation of the oligomer in the presence of the buffer. It has been suggested that pure water has the ability to wrap around aggregates, which, in buffer, could lead to insoluble aggregates [38], facilitating their monomerization and solubility. This effect can be easily and generally conceived of, considering that counterions in buffer solution screen the exposed charges of monomers, favoring their association, while in pure water, the absence of counterions maintains the electrostatic repulsion between monomers, favoring their conformation in isolation. Although we cannot rule out this effect in bsHPrK/P, we suggest that the reason for its oligomerization is the phosphate. The presence of phosphate would facilitate the self-association of the protein through its interaction with some arginine residues at the interface of the oligomer (Figure 5), as suggested by the X-ray structure of the homologous HPrP/K from *Staphylococcus xylosus* (PDB entry: 1KO7), where there are several salt-bridges in the interface [20]. Furthermore, the remaining solved X-ray structures of HPrK/P family members show the presence of a large number of electrostatic interactions in the interface among the different protomers of the oligomer, whose formation could also be triggered by the presence of the phosphate [19,20,21,22]. Therefore, solution bsHPrK/P is populated, as in the crystal structures of other HPrK/P proteins, by a hexameric species. The sequence identity between bsHPrK/P and the HPrK/P of *Staphylococcus xylosus* is 64%, while the identity with the HPrK/P of *Mycoplasma pneumoniae* is 34%. We used the structure of HPrK/P of *Staphylococcus xylosus* (instead of that of *Mycoplasma pneumoniae,* as in the SAXS calculations, Section 2.2) because: (i) it contains phosphate bound in the structure; (ii) it has a higher sequence identity; (iii) it has the same arginine residues as those conserved in bsHPrK/P, involved in such a binding interface (while the conservation of those arginine residues in HPrK/P of *Mycoplasma pneumoniae* is less clear). We speculate (from our DLS experiments in other buffers) that phosphate is probably not the sole anion to trigger self-association, but others could also favor the oligomer species formation. Finally, the specific role of phosphate ions in the oligomeric equilibria in solution is suggested by the Kratky plots reported in Figure S4. It is evident that, in the sodium phosphate buffer and Tris buffer (Figure S4C), protein concentration affects oligomeric equilibria in different ways.

### 3.2. The pH Conformational Changes of bsHPrK/P in Solution

Our studies show that the protein acquired a native-like structure in a narrow pH range (from around 7.0 to 8.0) since, at pH values below pH 7.0, the protein precipitated (as shown by fluorescence, CD, and DLS experiments), and, at acidic pH values (smaller than 3.5), the protein seemed to populate a molten-globule-like conformation [25]. These results indicate that the protein was very pH-sensitive around physiological pH. At pH values above pH 8.0, bsHPrK/P showed a titration; although, at this stage, we cannot rule out the titration of other residues (Arg and/or Lys), the fact that this transition was observed after fluorescence excitation at 280 nm indicates that it is due to some, if not all, of its five tyrosine residues [26,27].

The narrow pH range (from around 7.0 to 8.0) in which bsHPrK/P acquired a native-like structure would ensure that it is only functional under physiological conditions. However, although the protein acquired a native-like structure between pH 7.0 and 8.0, there were subtle conformational changes in that pH range. Furthermore, it had the largest stability at pH 7.0, as measured by the *T*_m_. Since the protein is an oligomer at those pHs, we should expect: (i) if dissociation and monomer unfolding occurred concomitantly, a concentration-dependent shift in the sole apparent *T*_m_ value as the protein concentration was raised; (ii) several transitions indicating oligomer dissociation and monomer unfolding. We did not observe an increase in the thermal denaturation midpoint when the protomer concentration was raised (in either intrinsic fluorescence (Figure S2) or far-UV CD at most of the pH values (Figure S3)); however, we did observe two transitions in the far-UV CD thermal denaturations at pH 7.6, the first of which had a protein-concentration-dependent *T*_m_ (Figure S3), and the second one was protein-concentration-independent. This result indicates that the thermal unfolding of bsHPrK/P was not two-state [37]. In addition, when comparing the thermal unfolding results obtained by CD with those obtained by fluorescence, we can conclude that either (i) fluorescence was spectroscopically silent to dissociation, or (ii) oligomer dissociation had a similar thermal denaturation midpoint to that of monomer unfolding, and both sigmoidal curves overlapped. The fact that ANS-followed thermal denaturation at 0 M GdmCl (Table 3) yielded a slightly lower thermal midpoint than the intrinsic-fluorescence-followed thermal denaturations points to the second possible explanation, where dissociation would leave a large amount of solvent-exposed hydrophobic regions, as indicated in the X-ray structures of the homologous proteins [19,20,21,22]. Alternatively, we could think that the affinity constant of self-association was very small, and then, as has been observed in other oligomeric proteins [40], we would not observe the protein-concentration-dependence of *T*_m_ in the range of protein concentrations used. However, if this assumption was right, we should not observe any protein concentration-dependence in the chemical denaturations (Figure 4). Finally, one could offer another reason for the lack of protein concentration-dependence in the observed *T*_m_ due to the irreversibility of the denaturation process; it has been proven in the highly irreversible thermal unfolding of other hexameric proteins [41] that the lack of such dependence is due to the fact that the dissociation step follows the rate-limiting step (which is present because the unfolding is kinetically controlled).

It was observed that, at pH 7.0 and 8.0, the thermal denaturation midpoints obtained by intrinsic fluorescence were slightly higher than those measured by CD. We have reported similar behavior for other large monomeric proteins which unfold irreversibly, by following the changes with the same two spectroscopic probes [42,43]. Such behavior has been explained as being due to the large irreversibility of the process, which is probably increased at the high basic pH values where thermal denaturations occurred. To the best of our knowledge, there are not many hexameric proteins whose unfolding has been studied by heat; in the example of nucleoside diphosphate kinase (NDPK), thermal unfolding involved a coupled dissociation-unfolding mechanism, with a thermal denaturation midpoint of ~60 °C [44,45]. This value is similar to that observed in this work for dissociation (Section 2.1.1 and Section 2.1.2), suggesting that the isolated monomers of bsHPrK/P could have a large stability. Other examples further indicate the concomitant dissociation and monomer unfolding by either temperature or chemical denaturants [46,47].

The fact that we observed two transitions in the thermal denaturation followed by far-UV CD at pH 7.6 indicates that: (i) oligomer dissociation by temperature (probably involving the equilibrium: (bsHPrK/P)_6_ ↔ 6 (bsHPrK/P), since the second transition was protein concentration-independent) did not cause the loss of all secondary structure in the monomers; (ii) isolated monomers can exist above 65 °C (Figure S3) with some residual structure. In addition, the fact that oligomer dissociation had a high *T*_m_ value indicates that a substantial amount of the stability of the protein must come from inter-subunit interactions.

### 3.3. The Equilibrium Unfolding of bsHPrK/P by Urea and GdmCl

Since chemical denaturations were irreversible, we could not obtain a value of the ∆*G* of unfolding, but we can qualitatively discuss the [denaturant]_1/2_-and *m*-values for both denaturants. The *m*-value is the slope of the variation in the free energy of unfolding with the denaturant concentration (urea or GdmCl), and [denaturant]_1/2_- value is the concentration of denaturant at which the fraction of unfolded protein equals 0.5.

Denaturation followed by any of these denaturants led to different results and, therefore, to different unfolding equilibrium pathways. While the urea unfolding involved a concomitant dissociation and unfolding of monomer, as indicated by intrinsic fluorescence and CD, the GdmCl denaturation indicated the presence of several intermediates, with one of them (populated at 1 M GdmCl) oligomeric in nature (Figure S6B and Table 2). The differences must rely on the non-ionic nature of urea when compared to the salt, GdmCl. Oligomerization has been shown to be affected by NaCl concentration in other proteins [48,49,50], and the conclusion we can obtain in bsHPrK/P is that electrostatic interactions must affect oligomerization (as is pinpointed by the X-ray structures of homologous HPrK/P proteins [19,20,21,22]).

The urea denaturations were protein concentration-independent by far-UV CD and fluorescence (Table 2). Since the protein is an oligomer, there are several possible explanations for this behavior. First, both techniques were spectroscopically silent to dissociation, and we were only monitoring monomer unfolding; since far-UV CD reports on dissociation when the protein was heated (Figure S3), we believe that it would be strange for this technique to not report on protein dissociation in the presence of urea. Second, the self-association constant is very high, as discussed above (Section 3.2.). Third, dissociation had a similar [urea]_1/2_–value to that of monomer unfolding (as happened with the *T*_m_ in thermal denaturations), and the two sigmoidal curves were very close, yielding an apparent single sigmoidal curve, with a very small *m*-value (i.e., poor cooperativity [49,50]). We favor the latter explanation, based on the evidence of urea denaturations monitored by ANS (Table 2). The ANS-followed urea denaturations showed a slightly smaller [urea]_1/2_-value (3.7 vs. 4.0 M at 1.8 µM and 5.8 µM protomer concentration) (Table 2 and Figure 4A) than those monitored by far-UV CD and intrinsic fluorescence, suggesting that ANS probably mainly (but not exclusively) reported on the oligomer dissociation.

GdmCl denaturations reported at least two transitions (Figure 4B). The first one, occurring between 0 and 1 M GdmCl, could not be clearly assigned to a dissociation step (due to the scattering of experimental data in any of the three techniques, Figure 4B), but the resulting species at 1 M GdmCl was an oligomeric one, as shown by the protein concentration-dependence of the second transition observed at higher denaturant concentrations (Table 2). Alternatively, this first transition could be due to conformational rearrangements of the hexameric species, which did not result in an increase in protein stability (Table 3) due to the Gdm^+^ cation, as has been shown to occur in other oligomeric proteins at low GdmCl concentrations [49]. This behavior at low GdmCl concentrationshas been observed in dimeric proteins [51], and it has been attributed to the presence of oligomeric intermediates. The second transition observed in GdmCl denaturations involved concomitant oligomer dissociation and monomer unfolding (Table 2). The fact that, once again, ANS-followed GdmCl denaturations had a smaller, proteinconcentration-dependent [GdmCl]_1/2_-value (2.6 vs. 3.2 M at 1.8 µM protomer concentration, Table 2), than those followed by fluorescence of far-UV CD for this second transition indicates that ANS reported mainly on the oligomer dissociation of the intermediate species populated at 1 M GdmCl.

## 4. Materials and Methods

### 4.1. Materials

Ampicillin and isopropyl-β-D-1-thiogalactopyranoside were obtained from Apollo Scientific (Stockport, UK). Imidazole, Trizma base, DNAse, SIGMAFAST protease tablets and His-Select HF nickel resin were from Sigma-Aldrich (Madrid, Spain). Ultra-pure GdmCl and urea were from Pierce (USA). Amicon centrifugal devices with a cut-off molecular weight of 10 kDa were from Millipore (Barcelona, Spain). The rest of the used materials were of analytical grade. Water was de-ionised and purified on a Millipore system.

### 4.2. Protein Expression and Purification

The vector containing bsHPrK/P was a kind gift from Dr. A. Galinier. It contains a His-tag to allow for protein purification. Expression and purification of the protein were carried out as previously described [23] in the *E. coli* BL21 (DE3) strain, with the use of a SIGMAFAST protease tablet per 5 L of culture during the lysis step. After elution from the Ni-resin, the protein was concentrated by using Amicon centrifugal devices at 5 °C, and an additional polishing gel filtration column step was carried out. The concentrated sample was loaded onto a Superdex 200 16/60 gel filtration column connected to an AKTA-FPLC System (GE Healthcare Life Sciences, Barcelona, Spain), using s 20 mM Tris buffer (pH 7.5) with 150 mM NaCl as running buffer, and following the absorbance at 280 nm. In this column, and under these buffer conditions, the protein always eluted as a wide peak in the range volume from 70 to 80 mL (the void volume of such column is 43 mL and the bed volume was 119 mL, as determined by changes in the conductivity). The fractions containing bsHPrK/P were pooled and concentrated using Amicon centrifugal devices at 5 °C. Protein was dialysed against water for12 h with four changes, and stored, after being flash-frozen, at −20 °C until use. Dialyzed samples had a pH value between 7.2 and 7.4, depending of the concentration of the protein in the particular stock.

The peak containing the protein eluted from the semi-preparative column was loaded into an analytical column Superdex 200 HR 10/30 on the same chromatography instrument and using the same buffer. The protein was eluted as a series of peaks, which could not be fully resolved between 8.20 and 9.30 mL, and a small shoulder at 6.8 mL. The void volume of this column was 6.47 mL and the bed volume was 19.31 mL, as determined by changes in conductivity. Therefore, we were not able to separate the different oligomeric species present in the buffer solution to study them in isolation.

Protein concentration was determined from the absorbance, at 280 nm, of its five tyrosines (Tyr43, Tyr46, Tyr47, Tyr145 and Tyr241) and single tryptophan (Trp235) [52].

### 4.3. Fluorescence

#### 4.3.1. Acquisition of Fluorescence Spectra

Fluorescence spectra were collected on a Cary Varian spectrofluorometer (Agilent, Santa Clara, CA, USA), interfaced with a Peltier unit. All experiments were carried out at 25 °C. Following the standard protocols used in our laboratories, the samples were prepared the day before and left overnight at 5 °C; before experiments, samples were left for 1 h at 25 °C. A 1-cm-pathlength quartz cell (Hellma, Kruibeke, Belgium) was used.

For the pH denaturation experiments, protein samples were excited at 280 and 295 nm in the pH range from 2.0 to 14.0. Slit widths were 5 nm. Sample concentrations were 1, 1.8, 10 and 15 µM (in protomer units, see Section 2.2). The other experimental parameters and the buffers used have been described elsewhere [53]. Briefly, final buffer concentration was 50 mM in all cases, and the corresponding salts and acids used were: pH 2.0–3.0, phosphoric acid; pH 3.0–4.0, formic acid; pH 4.0–5.5, acetic acid; pH 6.0–7.0, NaH_2_PO_4_; pH 7.5–9.0, Tris acid; pH 9.5–11.0, Na_2_CO_3_; pH 11.5–13.0, Na_3_PO_4_. Appropriate blank corrections were made in all spectra. The pH of each sample was measured after completion of pH-denaturations with an ultra-thin Aldrich electrode in a Radiometer (Copenhagen, Denmark) pH-meter. In the pH denaturation experiments, protein precipitation was always observed between pH 3.5 and 6.5. Samples at those pH values were centrifuged to remove the precipitate.

Chemical denaturations at pH 7.0 (in 50 mM sodium phosphate buffer), followed by either intrinsic or ANS (see Section 2.2) fluorescence, and far-UV CD (see below), were carried out by dilution of the proper amount of a 7 M GdmCl or 8 M urea stock solutions. The GdmCl or urea concentrations in the stock solutions were quantified by using refractive index measurements [54]. For the GdmCl denaturations, the protein concentrations were 1.8, 6 and 12.6 µM (in protomer units); for the urea denaturations, we used 1.8, 6, 11 and 18 µM (in protomer units). Both the chemical and pH denaturations at the different explored protein concentrations were repeated at least three times with new samples. Variations using the same voltage in the fluorescence photomultiplier from repeated day-to-day experiments were 3–5%.

Refolding experiments were carried out by exchanging a concentrated stock solution of bsHPrK/P (~70 µM) in 7 M GdmCl or 8 M urea, by the use of Amicon Centrifgugal devices. The protein was then diluted in the proper amount of water to yield the desired GdmCl concentration and the corresponding amount of buffer was added. Experiments were carried out (either by fluorescence or CD, see below) at 25 °C in sodium phosphate buffer (50 mM, pH 7.0) with the same experimental set-up as described above. Final protein concentration in these refolding experiments was 1.8 µM (for either urea or GdmCl).

#### 4.3.2. Thermal Denaturations

Experiments were performed at constant heating rates of 60 °C h^−1^ with an average time of 1 s, and a temperature step of 0.2 °C. The “average time” is the “sampling time” of the instrument (Cay Varian spectrofluorometer) at each temperature. In a thermal scan experiment, this value should be lower than the experimental scan rate, to ensure that the temperature is constant during the acquisition of fluorescence emission at a particular temperature. Protein concentrations were 11 µM in each thermal denaturation. Thermal scans were collected at 315, 330 and 350 nm after excitation at either 280 or 295 nm, typically from 25 to 80 °C, at different pH values in the buffers described above. The rest of the experimental set-up was that described above. Irreversibility was tested by acquiring spectra after thermal denaturation, and comparing their shape and intensity with those of the spectra acquired before heating at the same temperature. All the thermal denaturations were irreversible; the apparent thermal denaturation midpoint was estimated from a two-state equilibrium equation (see Section 4.7).

#### 4.3.3. 1-Anilino-8-Naphtalene Sulfonate (ANS) Binding

The excitation wavelength was 370 nm, and emission was measured from 400 to 600 nm at 25 °C. Slit widths were 5 nm for both excitation and emission. ANS stock solution (10 mM) was prepared in water and diluted to yield a final concentration of 100 µM in each sample, with a final protein concentration of 1.8 µM (either in the pH, urea and GdmCl denaturation experiments). An additional experiment with 5.8 µM of protein was carried out in the presence of urea or GdmCl. Buffer concentrations were the same as used in the intrinsic fluorescence experiments. Spectra from blank solutions were subtracted from the corresponding spectra. In the pH and chemical denaturations, the pH of each sample was measured after completion of titration with an ultra-thin Aldrich electrode in a Radiometer pH-meter. The protein was precipitated for pH values between 3.5 and 6.5 in the pH denaturation experiments, even in the presence of ANS. In these samples, the solution was centrifuged to remove the precipitate.

We also used ANS to follow thermal denaturations at a protein concentration of 1.8 µM at different concentrations of GdmCl, as this can provide information on how the different solvent-exposed hydrophobic patches change their environment upon heating [55]. The experimental set-up in these denaturations was the same as was used in the intrinsic fluorescence, except for the excitation (370 nm) and emission (480 nm) wavelengths. Thermal denaturations were always irreversible.

#### 4.3.4. Quenching Experiments

To further characterize the solvent-exposure of the sole tryptophan of bsHPrK/P (Trp235) and its five tyrosines, we carried out quenching experiments with either acrylamide (in the range from 0 to 0.6 M) or KI (in the same concentration range). The experiments with KI were carried out in the presence of: (i) 100 mM Na_2_S_2_O_3_ to prevent I_3_^−^ formation, which would quench tryptophan fluorescence emission; (ii) a constant ionic strength of 700 mM by the use of KCl. The bsHPK/P concentration was always 1.8 µM (in protomer units). Experiments were acquired with the same experimental set-up as in the acquisition of the fluorescence spectra at 25 °C at 7.0 (50 mM, sodium phosphate buffer) and pH 8.4 (50 mM, Tris buffer). Experiments were also carried out for both quenching agents at 5 M GdmCl at pH 7.0 (50 mM, sodium phosphate buffer). The quenching plots with acrylamide, obtained by excitation at 280 nm, showed an exponential behavior at the four experimental conditions. They were fitted to [56].

(1) $$\frac{I_{0}}{I}=\left(1+K_{SV}\left[acrylamide\right]\right)e^{\left(\upsilon^{}\left[acrylamide\right]\right)}$$

where *I*_0_ is the fluorescence intensity at any selected wavelength when no acrylamide was added; *I* is the intensity at the same wavelength when a particular concentration of acrylamide, [acrylamide], was present; *K*_sv_ is the collisional Stern–Volmer constant; υ is the static quenching constant. On the other hand, quenching by KI, by excitation at 280 or 295 nm, or that of acrylamide obtained by excitation at 295 nm yielded linear plots, and then, the linear form of Equation (1) was used [56].

(2) $$\frac{I_{0}}{I}=1+K_{SV}\left[KI\right]$$

### 4.4. Far UV Circular Dichroism (Far-UV CD)

The far-UV CD spectra were collected on a Jasco J810 spectropolarimeter (Jasco, Tokyo, Japan) with a thermostated cell holder, and interfaced with a Peltier unit at 25 °C. The instrument was periodically calibrated with (+)-10-camphorsulphonic acid. A path length cell of 0.1 cm was used (Hellma, Kruibeke, Belgium). All spectra were corrected by subtracting the corresponding baseline. The concentration of bsHPrK/P was the same used in the fluorescence experiments.

#### 4.4.1. Far-UV CD Spectra

Isothermal wavelength spectra at different pH values, urea and GdmCl concentrations were acquired at a scan speed of 50 nm min^−1^ with a response time of 2 s, a band-width of 1 nm, and averaged over six scans from 205 to 250 nm. Protein concentrations were the same as used in fluorescence. Both the chemical and pH denaturations were repeated at least three times with new samples. Buffer concentrations for the pH- and chemical-denaturation experiments were 50 mM, and the buffers were the same used in the fluorescence experiments. Day-to-day variations in the voltage of the photomultiplier, with the new prepared samples, were less than 4%. The samples were prepared the day before and left overnight at 5 °C to allow for equilibration. Before starting the experiments, samples were left for 1 h at 25 °C.

#### 4.4.2. Thermal Denaturations

Experiments were performed at constant heating rates of 60 °C h^−1^ and a response time of 8 s, with a temperature step of 0.2 °C and a bandwidth of 1 nm. Thermal scans were collected by following the changes in ellipticity at 222 nm, typically from 25 to 90 °C, at different pH values in the buffers described above. The rest of the experimental set-up was the same as reported in the acquisition of the far-UV CD spectra. No difference was observed between the scans aiming to test drifting spectropolarimeter signal. Thermal denaturations were always irreversible, as shown by: (i) the comparison of spectra before and after the heating at the same temperature; (ii) the changes in the voltage of the instrument detector [57]. The apparent thermal denaturation midpoint, *T*_m_, was estimated from a two-state equilibrium equation (Section 4.7).

### 4.5. Dynamic Light Scattering (DLS)

DLS measurements were performed in a Zetasizer Nano instrument (Malvern Instruments Ltd., UK) equipped with a 10 mW helium-neon laser (λ = 632.8 nm) and a thermoelectric temperature controller. Experiments were carried out at pH 7.0 in 50 mM, sodium phosphate buffer, and in water at different concentrations; at pH 6.5 in 50 mM MES buffer, only a single protein concentration (32 µM) was explored. Experiments were also carried out at pH 7.0 in 50 mM Tris buffer. All the experiments were performed at a fixed angle (Θ = 173°) at 25 °C, and analyzed with Zetasizer software V7.12 (Malvern Instruments Ltd., UK). Before each measurement, all the samples of bsHPrK/P were centrifuged for 30 min at 14,000× *g* and filtered in a 0.2 mm cut-off Millex filter to remove large aggregates and dust. Once in the cuvette, samples were sonicated for 1 min to remove bubbles. Each sample was measured 10 times, with 10 runs of 30 s each. The Z-average size was obtained by fitting the autocorrelation function with the cumulants method. The hydrodynamic radius, *R*_h_, and molecular weight (MW) were determined from the Stokes–Einstein equation, assuming a spherical shape for bsHPrK/P.

### 4.6. Small-Angle X-Ray Scattering (SAXS)

SAXS data were collected at the Austrian beamline at Elettra Synchrotron in Trieste, Italy [58]. Temperature was 25 °C during all measurements. Scattering patterns were recorded using the Pilatus 3 1 M detector system (Dectris, Switzerland). The transmitted X-ray beam was measured using a photodiode mounted on the beamstop. The 2D detector images were radially averaged, obtaining the scattering intensity as a function of the magnitude of the scattering vector *Q* defined as: *Q* = (4πsin(θ))/λ, where 2θ is the scattering angle, and λ = 0.154 nm, the X-ray wavelength. The incident and transmitted intensities were measured; data were corrected for sample transmission and fluctuations in the primary beam. The individual scattering patterns from all images of each sample were averaged, and the respective backgrounds, treated in the same way, were subtracted. The resulting scattering patterns were converted to absolute intensity by rescaling the forward intensity with BSA and water scattering. The sample stage was the µ-Drop sample changer, recently designed and developed in the Austrian beamline [59]. The µDrop system has several advantages over a capillary-based setup, the main of which is that, because just a single drop is placed, the used volume is 15 µL. Each measurement was performed on at least 10 injections of sample volumes of 15 µL, and was carried out four times for 20 s. Each SAXS spectrum acquisition was followed by 3 s of dead time. This approach allowed us to minimize the effects of both potential in-homogeneity and radiation damage in the protein sample. After SAXS data comparison, we averaged all the measured scattering data corresponding to the same nominal sample. Buffer measurements were always performed before and after sample measurements. SAXS curves were obtained from the stock solution in water at concentrations of 36, 18 and 8 µM for bsHPrK/P. Concentrations explored in the 50 mM sodium phosphate buffer were 36 and 8 µM.

### 4.7. Fitting of Chemical and Thermal Denaturations Followed by Spectroscopic Probes

To allow for a better comparison among the different probes used (intrinsic or ANS fluorescence), and since we can obtain information over the whole spectrum with the parameter, we also calculated the average energy, <λ>, which is defined as Equation (3) [60].

(3) $$\langle \lambda \rangle =\sum_{1}^{n}\left(\frac{1}{\lambda_{i}}I_{i}\right)/\sum_{1}^{n}I_{i}$$

where *I*_i_ is the fluorescence intensity at a particular wavelength, λ_i_.

The change in the physical property, *Y* (the intrinsic or ANS fluorescence intensity, or the ellipticity at 222 nm), for the thermal denaturations was fitted to

(4) $$Y=\left(Y_{N}+Y_{D}e^{\left(-\Delta G/RT\right)}\right)/\left(1+e^{\left(-\Delta G/RT\right)}\right)$$

where *Y*_N_ = α_N_ + β_N_[T] and *Y*_D_ = α_D_ + β_D_[T] are the baselines of the folded and unfolded states, respectively, for which a linear relationship with temperature is assumed; *R* is the gas constant; ∆*G* is the free energy of unfolding; *T* is the temperature in K.

Although the thermal denaturations, followed by either fluorescence (intrinsic or ANS) or CD, were irreversible, we obtained an apparent thermal denaturation midpoint, *T*_m_ (which is the temperature at which the fraction of unfolded protein equals 0.5). This value allows for an estimate of the stability of bsHPrK/P at the different pH values, if the thermal denaturations were reversible, from the change in the standard free energy of unfolding, ∆*G*, given by [32,33,34,35,36,61]

(5) ΔG(T)=ΔHm1−TTm−ΔCpTm−T+TlnTTm−RTln(1458Ct5)

where ∆*H*_m_ is the van’t Hoff unfolding enthalpy, *C*_t_ is the total concentration of protein expressed in hexamer equivalents, and ∆*C*_p_ is the heat capacity change in the unfolding reaction. In the numerator and denominator of Equation (4), it appears that Equation (5) does not impose restrictions on the value of the ∆*C*_p_ used in the fitting. The term $-RT\ln(1458C_{t}^{5})$ is obtained by taking the hexameric nature of the protein (Section 2.2) into account; thus, we have assumed that the main species when thermal unfolding was carried out was a hexamer.

For the urea or GdmCl denaturations (followed by intrinsic or ANS <λ> and ellipticity at 222 nm), the same Equation (4) was used, although the denaturations were also irreversible, and then, quantitative conclusions about the values of the determined thermodynamic parameters cannot be obtained. In this case, the free energy for chemical denaturations follows a linear relationship with denaturant concentration (∆*G* = m ([denaturant]_1/2_ -[denaturant]) $-RT\ln(1458C_{t}^{5})$), where *m*-value is the slope in the variation in the free energy of unfolding with the denaturant concentration (urea or GdmCl), and [denaturant]_1/2_ is the concentration of denaturant at which the fraction of unfolded protein equals 0.5 [61]), and the spectroscopic properties of the native and unfolded species are given by: Y_N_ = α_N_ + β_N_ [denaturant] and Y_D_ = α_D_ + β_D_[denaturant].

Fittings to Equations (4) and (5) were carried out by using the general curve-fit option of Kaleidagraph (Abelbeck Software, PA, USA).

## 5. Conclusions

We have described the conformation of a bsHPrK/P in solution. The protein was highly sensitive to pH changes. The protein appeared to be a hexamer at physiological pH, with an elongated shape. Chemical and thermal denaturations at physiological pHs indicate that unfolding of the protein occurred through several intermediates with different oligomerization states.

## Figures and Tables

**Table 1:** Quenching parameters in KI and acrylamide of bsHprK/P ^a^.

Conditions	Acrylamide b		KI	
	*K*_sv_ (M^−^^1^) (280) ^b^	*K*_sv_ (M^−^^1^) (295) ^c^	*K*_sv_ (M^−^^1^) (280)	*K*_sv_ (M^−^^1^) (295)
pH 7.0	1.1 ± 0.3 (4 ± 1)	1.6 ± 0.1	2.1 ± 0.1	1.8 ± 0.2
pH 8.0	6.3 ± 0.6 (1.8 ± 0.1)	1.6 ± 0.2	2.0 ± 0.1	1.7 ± 0.2
5 M GdmCl	15 ± 3 (2.2 ± 0.3)	4.8 ± 0.2	3.2 ± 0.3	3.0 ± 0.2

^a^ Errors are from fitting to Equation (1) (acrylamide, by exciting at 280 nm) and Equation (2) (KI, at both excitation wavelengths, and acrylamide by exciting at 295 nm). The *K*_sv_ values were obtained by fitting of fluorescence intensity at 330 nm at any condition versus concentration of quenching agent. Experiments were carried out at 25 °C. The quenching experiments in the presence of GdmCl were carried out at pH 7.0, 50 mM sodium phosphate buffer. ^b^ The value within the parenthesis is the ν (Equation (1)). ^c^ Acrylamide quenching resulted in a straight line at 295 nm (and then ν = 0).

**Table 2:** Chemical denaturations of bsHPrK/P ^a^.

	Urea			GdmCl d		
Biophysical Probe	Concentration (µM)	m (kcal mol−1M−1)	[urea]1/2 (M)	Concentration (µM)	m (kcal mol−1 M−1)	[GdmCl]1/2 (M)
<λ>^280 b^	1.8	1.1 ± 0.2	4.0 ± 0.2	1.8	2.6 ± 0.5	3.22 ± 0.05
	6	1.7 ± 0.2	3.97 ± 0.04	6	3.8 ± 0.4	3.38 ± 0.02
	11	1.8 ± 0.9	4.1 ± 0.2	12.6	5 ± 2	3.62 ± 0.06
	18	1.4 ± 0.3	4.1 ± 0.1			
<λ>^295^	1.8	1.0 ± 0.1	4.0 ± 0.3	1.8	3 ± 1	3.16 ± 0.07
	6	1.3 ± 0.8	4.0 ± 0.5	6	3.3 ± 0.3	3.38 ± 0.02
	11	1.4 ± 0.2	4.1 ± 0.3	12.6	7 ± 5	3.53 ± 0.08
	18	3.0 ± 1.6	3.9 ± 0.2			
<λ>^370^ (ANS)	1.8	1.5 ± 0.1	3.68 ± 0.04	1.8	1.7 ± 0.3	2.64 ± 0.08
	5.8	1.45 ± 0.09	3.7 ± 0.1	5.8	2.8 ± 0.9	3.14 ± 0.05
CD ^c^	1.8	1.1 ± 0.2	4.3 ± 0.2	1.8	1.9 ± 0.4	3.24 ± 0.08
	18	1.4 ± 0.2	4.4 ± 0.2	12.6	6 ± 2	3.62 ± 0.05

^a^*m* is the slope of the variation in the free energy of unfolding with the denaturant concentration (urea or GdmCl). Errors in the *m*- and [denaturant]_1/2_-values are fitting errors. Repetitions of the chemical-denaturations yielded differences of 0.3 kcal mol^−1^ M^−1^ in the *m*-values, and of 0.06 M in the [denaturant]_1/2_-values. All experiments were carried out at 25 °C and pH 7.0, 50 mM sodium phosphate buffer. The fluorescence and CD curves were fitted to Equation (4), taking the value of the free energy for a hexameric species into account. Reported bsHPrK/P concentration is in protomer units. ^b^ The <λ>^280^ indicates the <λ> obtained by excitation at 280 nm, and it has the same meaning for the corresponding <λ> with other superscripts. ^c^ The values were obtained by following the changes in the raw ellipticity at 222 nm. ^d^ The values reported for the GdmCl denaturations are those for the second transition.

**Table 3:** Apparent thermal unfolding temperatures (in °C) of bsHPrK/P at different GdmCl concentrations.

[GdmCl] (M)	I_330_ (280) ^a^	I_480_ (370) ^b^
0	71.83 ± 0.07	69.13 ± 0.07
0.25	59.5 ± 0.2	66.30 ± 0.06
0.5	57.7 ± 0.3	60.2 ± 0.2
0.75	54.8 ± 0.2	54.4 ± 0.2

^a^ It was obtained from the fitting of the sigmoidal curve observing the intensity at 330 nm after exciting at 280 nm. Errors are fitting errors to Equation (4), where the free energy is given by Equation (5). Experiments were carried out at 6 µM (protomer units). ^b^ This was obtained from the fitting of the sigmoidal curve observing the intensity at 480 nm after exciting at 370 nm. Errors are fitting errors to Equation (4), where the free energy is given by Equation (5). Experiments were carried out at 1.8 µM (protomer units).

## Data Availability

All the materials are available from the authors upon reasonable request.

## Supplementary Materials

The following are available online at https://www.mdpi.com/1422-0067/22/6/3231/s1: there are six figures in the Supplementary Material (S1 to S6): fluorescence spectrum of bsHPrK/P and far-UV CD spectrum at physiological pH (Figure S1); thermal denaturations followed by fluorescence at different pH values (Figure S2); thermal denaturations followed by far-UV CD at different pH values (Figure S3); the Kratky plots of the SAXS measurements (Figure S4); refolding experiments in urea (followed by far-UV CD) and GdmCl (followed by <λ> after excitation at 280 and 295 nm) of bsHPrK/P (Figure S5); unfolding experiments at two concentrations of protein mapped by the <λ> after excitation at 280 and 295 nm (Figure S6).

## Abbreviations

ANS	1-anilino-8-naphtalene sulfonate
CD	Circular dichroism
GdmCl	Guanidinium hydrochloride
HPr	Histidine phosphocarrier
HPrK/P	HPr kinase/phosphorylase
bsHPrK/P	HPr kinase/phosphorylase from *Bacillus subtilis*
MW	Molecular weight
NDP	Nucleoside diphosphate kinase
PEP	Phosphoenolpyruvate
PTS	Phosphotransferase system
SAXS	Small angle X-ray scattering
UV	Ultraviolet
