# Supplementary material for: The Histidine Phosphocarrier Kinase/Phosphorylase from Bacillus Subtilis Is an Oligomer in Solution with a High Thermal Stability
Source: Int J Mol Sci. 2021 Mar 22;22(6):3231. doi: 10.3390/ijms22063231 (PMC8004850; doi:10.3390/ijms22063231)
Supplement: Supplementary file 1 [file ijms-22-03231-s001.pdf]

Supplementary Information for the manuscript

The histidine phosphocarrier kinase/phosphorylase from *Bacillus subtilis* is an oligomer in solution with a high thermal stability

José L Neira, Ana Cámara-Artigas, Jose Ginés Hernandez-Cifre and María Grazia Ortore

SUPPLEMENTARY FIGURE LEGENDS

Figure S1: **Fluorescence and far-UV CD spectra of bsHPrK/P**: Fluorescence spectrum obtained by excitation at 280 nm (A), and far-UV CD spectrum (B) of the isolated protein at 25 ºC in phosphate buffer (50 mM, pH 7.0).

Figure S2: **Thermal denaturations of bsHPrK/P followed by changes in intrinsic fluorescence intensity at different pH values**: (A) At low protomer concentration, and (B) at high protomer concentration in the pH range from 7.0 to 8.0.

Figure S3: **Thermal denaturations of bsHPrK/P followed by changes in ellipticity at 222 nm at different pH values**: (A) At 3.6 M of protomer concentration. (B) At 1.8 M of protomer concentration in the pH range from 7.0 to 8.0.

Figure S4: **Kratky plots of SAXS data obtained for bsHPrK/P in different buffer solutions**: Kratky plots resulting from SAXS curves normalized according to their

concentration: (A) in pure water (39 and 8 M for violet circles, and green squares, respectively); (B) dissolved in 50 mM sodium phosphate buffer (pH 7.0) (35 and 8 M for violet circles and green squares, respectively), (C) dissolved in Tris buffer 50 mM (pH 7.0) (35 and 8 M for violet circles and green squares, respectively).

Figure S5: **Reversibility of urea and GdmCl denaturations followed by far-UV CD and fluorescence:** (A) Urea refolding followed by the changes in ^{222}. (B) GdmCl refolding followed by the changes in < > after excitation at 280 or 295 nm. Experiments were carried out at 25 ºC in phosphate buffer (50 mM, pH 7.0).

Figure S6: **GdmCl denaturations of HPrK/P at different protomer concentrations**: (A) Changes of the intrinsic fluorescence monitored by the < >, after excitation at 280 at two different concentrations (the experimental data for the highest protomer concentration are the real average energy values showed in main text normalized to allow for comparison of < >^{ANS} in Fig. 4 of the main text). (B) Changes of the intrinsic fluorescence monitored by the < >, after excitation at 295 at two different concentrations Experiments were carried out at 25 ºC in phosphate buffer (50 mM, pH 7.0). The errors in the calculated < > are estimated to be less than 1 % from the intensity measurements at different days in the repeated experiments.

Fig. S1

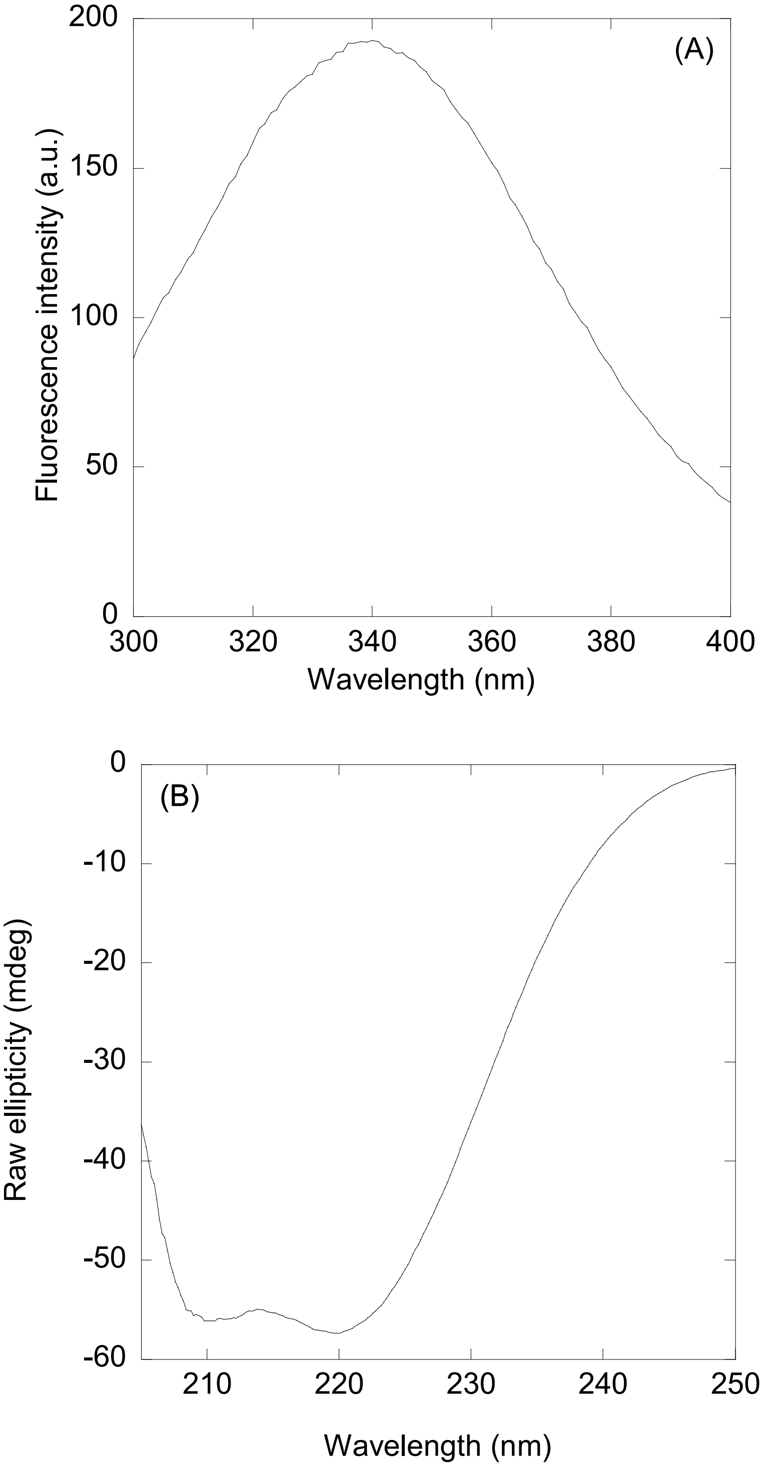
(A)
Fluorescence intensity (a.u.)
200
150
100
50
0
300
320
340
360
380
400
Wavelength (nm)
(B)
Raw ellipticity (mdeg)
0
-10
-20
-30
-40
-50
-60
210
220
230
240
250
Wavelength (nm)

Fig. S2

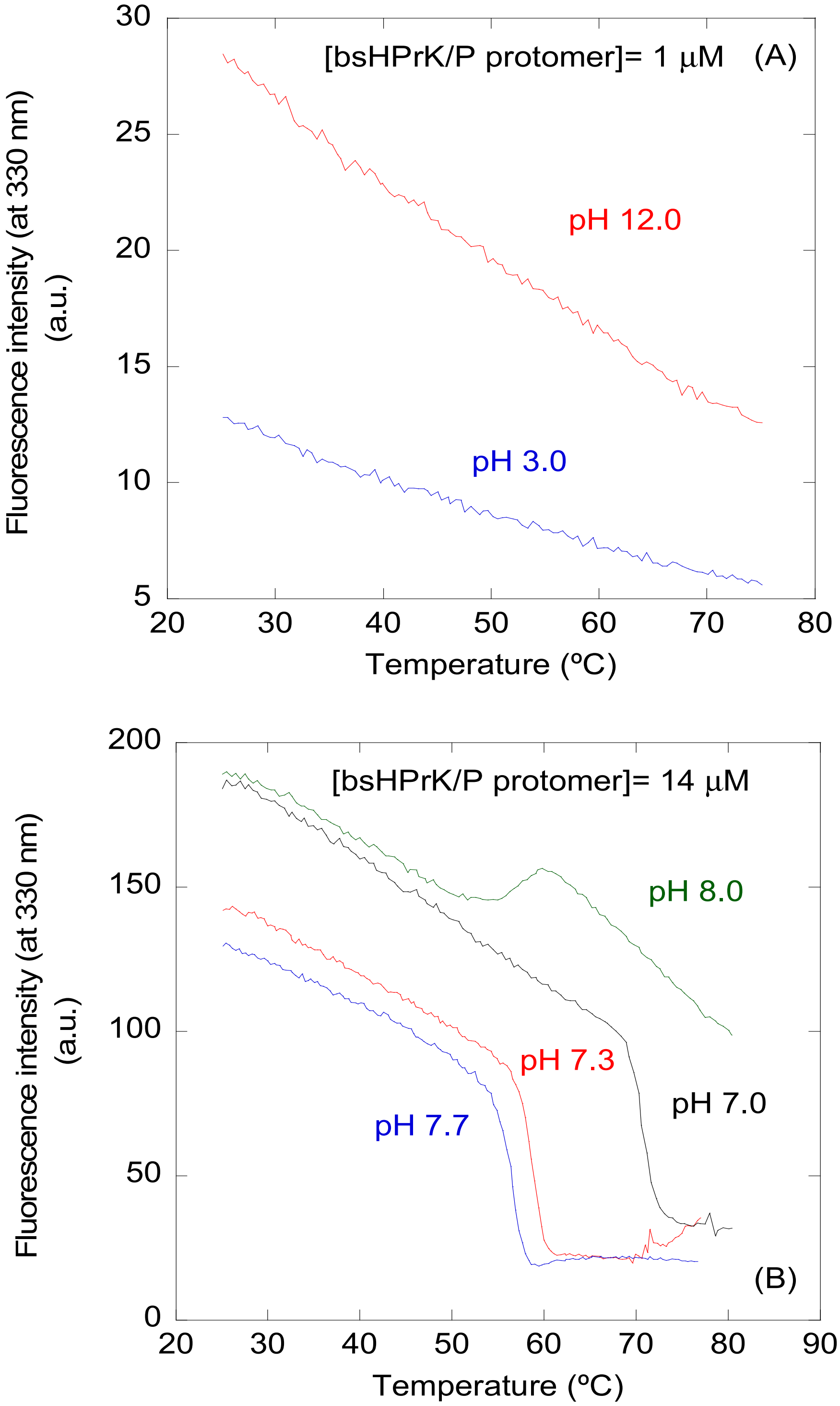

[bsHPrK/P protomer]= 1 μM
(A)
pH 12.0
pH 3.0
Fluorescence intensity (at 330 nm) (a.u.)
Temperature (ºC)
30
25
20
15
10
5
20
30
40
50
60
70
80
[bsHPrK/P protomer]= 14 μM
(B)
pH 8.0
pH 7.3
pH 7.0
pH 7.7
Fluorescence intensity (at 330 nm) (a.u.)
Temperature (ºC)
200
150
100
50
0
20
30
40
50
60
70
80
90

Fig. S3

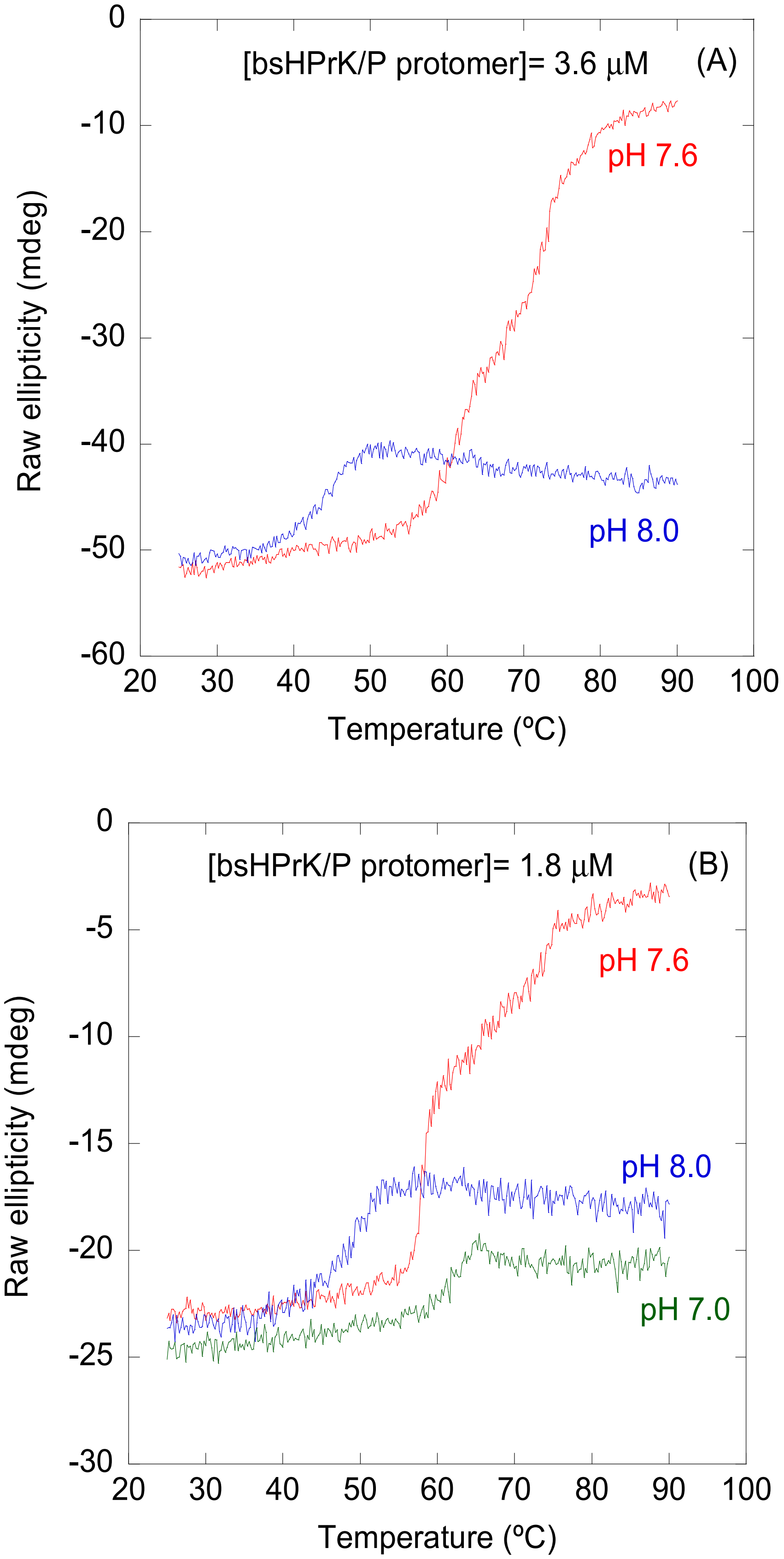
[bsHPrK/P protomer]= 3.6 µM
(A)
pH 7.6
pH 8.0
Raw ellipticity (mdeg)
Temperature (ºC)
0
-10
-20
-30
-40
-50
-60
20
30
40
50
60
70
80
90
100
[bsHPrK/P protomer]= 1.8 µM
(B)
pH 7.6
pH 8.0
pH 7.0
Raw ellipticity (mdeg)
Temperature (ºC)
0
-5
-10
-15
-20
-25
-30
20
30
40
50
60
70
80
90
100

Fig. S4

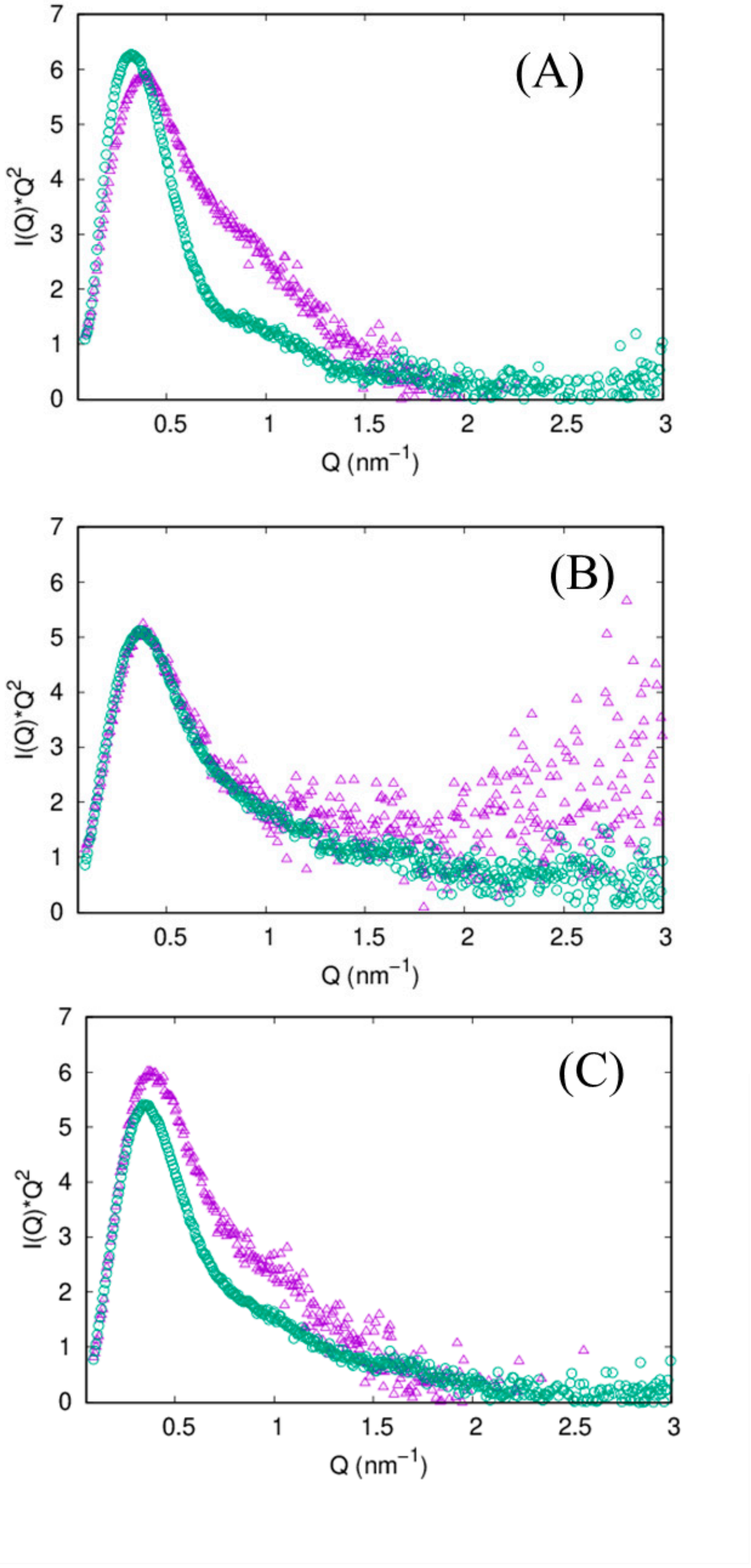

Fig. S5

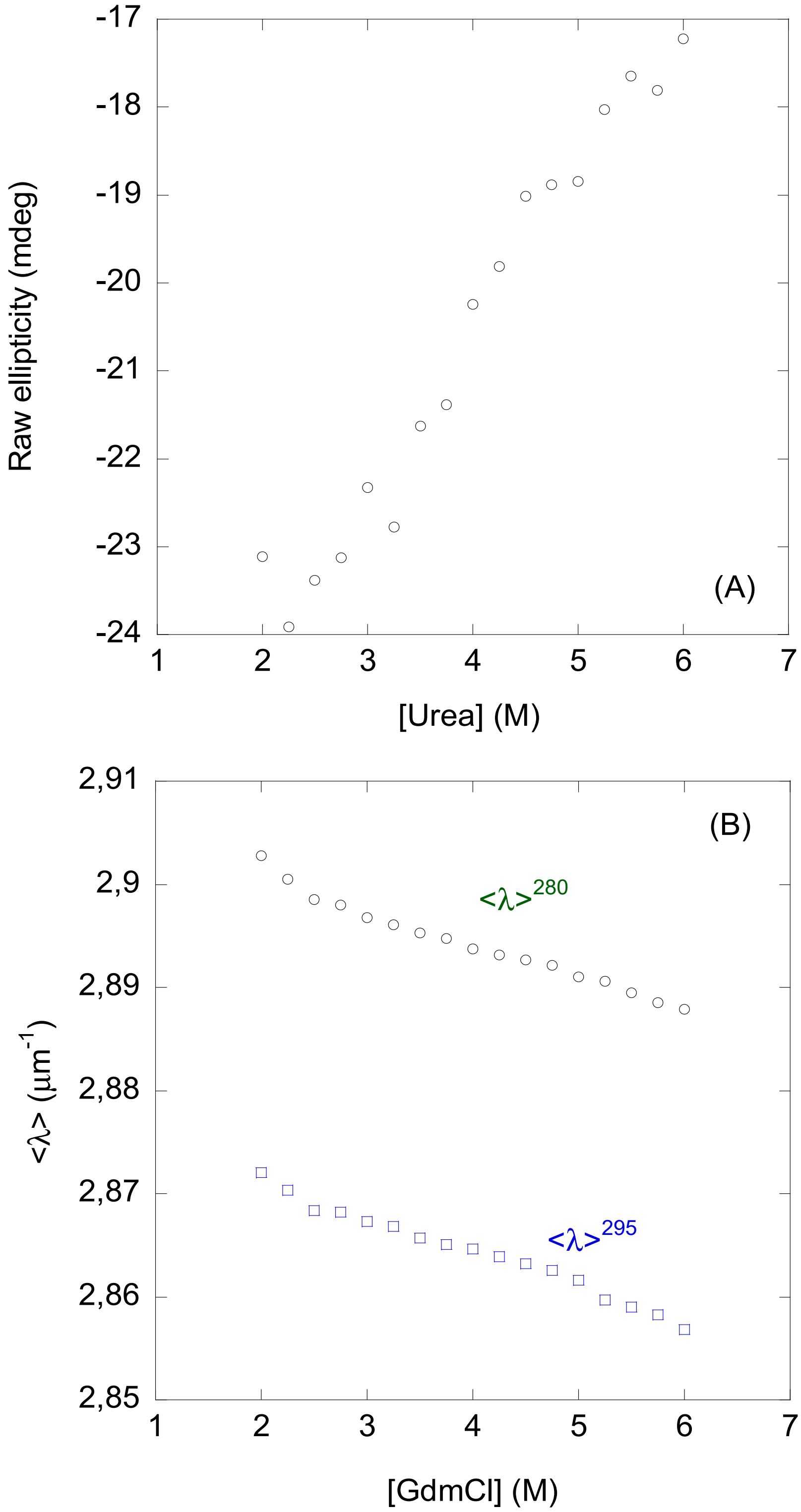

-17
-18
-19
-20
-21
-22
-23
-24
Raw ellipticity (mdeg)
(A)
1 2 3 4 5 6 7
[Urea] (M)
2,91
2,9
2,89
2,88
2,87
2,86
2,85
<λ> (μm-1)
(B)
<λ>280
<λ>295
1 2 3 4 5 6 7
[GdmCl] (M)

Fig. S6

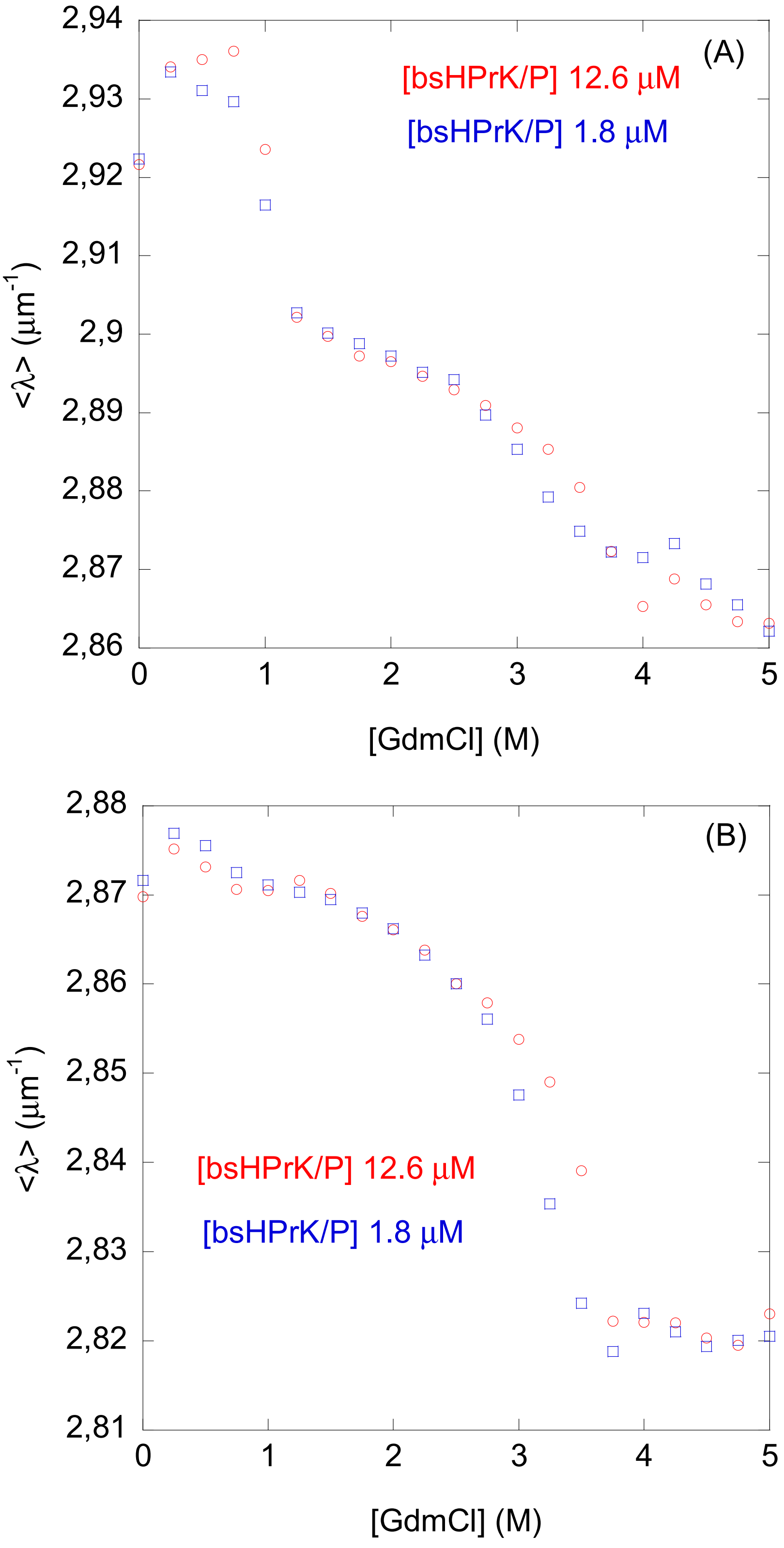
(A)
[bsHPrK/P] 12.6 µM
[bsHPrK/P] 1.8 µM
<λ> (µm-1)
[GdmCl] (M)
(B)
[bsHPrK/P] 12.6 µM
[bsHPrK/P] 1.8 µM
<λ> (µm-1)
[GdmCl] (M)
